# Automated concrete crack detection enhanced by deep learning and generative adversarial networks

**DOI:** 10.1371/journal.pone.0354018

**Published:** 2026-07-20

**Authors:** Yue Chang

**Affiliations:** Liaoning Provincial Transportation Planning and Design Institute Co., Ltd., Liaoning, China; Henan Polytechnic University, CHINA

## Abstract

Cracks on the surface of concrete structures are an obvious indication of their condition. However, traditional visual inspection methods are significantly influenced by human factors. They are accurate for larger cracks. However, they introduce subjective judgment when detecting smaller cracks. They also pose safety risks. Automated detection methods face challenges in achieving lightweight implementation due to limited annotated data and difficulties in complex environments. This paper proposes a method for detecting and measuring concrete cracks. The method uses a generative adversarial network (GAN) to augment data and a U-Net segmentation network. Conditional GAN (cGAN) is proposed to generate high-fidelity crack images using training-set data only, avoiding data leakage and effectively solving insufficient sample issues. A U-Net segmentation network that has been enhanced performs crack segmentation. This network incorporates channel attention modules and dilated residual blocks. A hybrid loss function that combines the Dice coefficient and binary cross-entropy resolves class imbalance. Fractal theory quantifies the geometric features of the detected cracks. The crack segmentation model that was trained on cGAN-augmented datasets achieved an average precision (AP) of 82.03% and an F1 score of 83.24%, as demonstrated by experimental results. The fractal dimension–based crack measurement method achieves an accuracy of ≤4% error for complex crack networks (fractal dimension >1.6). The proposed method provides a reproducible, automated, high-precision solution for concrete crack detection and quantification, with verified stability and engineering applicability.

## 1 Introduction

As infrastructure’s service life increases and environmental effects accumulate, it inevitably experiences damage such as cracking, corrosion, and fatigue [1,2]. Cracking is one of the most common types of damage to infrastructure. It is an early warning sign of structural safety hazards, and its formation and progression, driven by factors such as design, loading, and environment, can lead to issues like leakage, material degradation, reduced load-bearing capacity, and safety incidents [3,4]. From a mechanical perspective, the shape and location of cracks offer valuable information about structural stress conditions, stiffness degradation, and causes of damage. Accurately determining the position, size, morphology, and development trend of a crack enables inspection personnel to better assess structural health, formulate appropriate maintenance plans, ensure operational safety, and extend service life [5].

Current crack detection relies entirely on manual inspections using ladders, telescopes, and bridge inspection vehicles. Although direct, this method is susceptible to human error and false positives. Inspections are particularly challenging in high-altitude areas, near bodies of water, and at tunnel crowns and waistlines. The mental and physical condition of inspectors can impact results, leading to low efficiency and high costs, as well as the potential for overlooking details and misidentifying defects. Digital image processing technology can replace manual inspections, achieve automation and remote detection and improving efficiency. However, due to its limited generalization capability, the technology’s accuracy significantly decreases in complex backgrounds or environments with high illumination variability (e.g., tunnels). Furthermore, substantial manual calibration is required to achieve acceptable performance levels, which makes its practical application in engineering challenging [6]. Advances in machine vision technology have led to the widespread adoption of digital image processing algorithms for crack detection due to their automation and long-range capabilities. These algorithms can rapidly and accurately isolate cracks from image noise and backgrounds. However, these algorithms perform poorly in tunnel environments. Complex backgrounds and fluctuating lighting conditions compromise their accuracy. In practice, effective detection requires extensive expert analysis and manual parameter adjustments, which fail to meet tunnel lining crack detection requirements. The emergence of deep learning (DL)-based object detection algorithms offers a novel solution for tunnel lining crack detection [7]. These approaches learn crack features directly from the data, bypassing the need for predefined characteristics. Then, they utilize DL techniques and algorithms for crack identification. Current object detection algorithms based on deep learning include the SSD, YOLO, and RCNN families [7]. Many researchers have applied these algorithms to crack detection with significant success. For tunnel lining crack detection, deep learning–based algorithms demonstrate superior generalization capabilities and higher accuracy than traditional digital image processing methods. However, existing DL algorithms have two limitations: insufficient datasets and an imbalanced trade-off between model scale and accuracy. These limitations affect the effectiveness of deep learning algorithms in practical tunnel engineering inspections [8–11].

In recent years, object detection algorithms based on deep learning (DL), such as SSD, YOLO, and the R-CNN series, have demonstrated significant advantages [9,10]. These algorithms use an end-to-end approach that employs forward and backward propagation to autonomously learn optimal weights, eliminating the need for manually designed feature operators. Consequently, they exhibit superior environmental adaptability, detection accuracy, and robustness, while reducing the risks of manual inspection and false alarms. This makes them a key area of research at present. However, two major challenges persist in their practical implementation. (1) Accuracy issues arise because bridge cracks exhibit chaotic textures and varying dimensions. Predefined anchor boxes are not suitable for diverse requirements, resulting in missed detections and false positives. (2) Deployment feasibility: Much current work prioritizes accuracy improvements while neglecting lightweight modeling. This results in large-scale models that are unsuitable for resource-constrained edge devices. Alternative methods, such as radar, laser scanning, infrared, and acoustic sensing, are also excluded due to drawbacks like high cost, low sensitivity, time-consuming processing, significant environmental susceptibility, and noise interference.

This paper proposes a concrete method for detecting and analyzing cracks based on training-set only cGAN data augmentation data augmentation and an enhanced U-Net segmentation network to address these challenges. The paper’s main contributions are threefold:

(1) A cGAN‑based data augmentation module trained exclusively on the training set. Unlike existing GAN augmentation methods that risk data leakage by using validation or test images, our cGAN is trained solely on training‑set samples. It generates high‑fidelity synthetic crack images with realistic textures and diverse backgrounds, effectively solving the small‑sample overfitting problem while strictly preventing information contamination.
(2) An enhanced U‑Net segmentation network with channel attention and dilated residual blocks. We propose a pixel‑level crack segmentation network that integrates channel attention mechanisms into skip connections to dynamically weight important features, and dilated residual blocks to enlarge receptive fields without losing spatial resolution. A dynamic Dice+BCE hybrid loss is designed to handle extreme class imbalance (crack pixels typically <5%), with the Dice weight α adaptively reduced from 0.7 to 0.4 during training to optimize boundary precision.
(3) A fractal box‑counting method for high‑precision geometric quantification. We apply fractal theory to quantify crack geometric parameters (area, length) from the segmented masks. The method achieves measurement error ≤4% for complex crack networks (fractal dimension >1.6) with confidence level >0.9, providing a reliable quantitative tool for structural health assessment. Compared with existing studies that use GAN or U‑Net in isolation, our framework uniquely integrates these three components into an end‑to‑end pipeline, with the training‑set‑only cGAN being a key methodological safeguard often overlooked in prior work.

## 2 Related work

### 2.1 Traditional crack detection methods

Early crack detection relied on digital image processing techniques. Edge detection algorithms, such as Sobel, Canny, and Laplacian, identified gray-level gradient changes in images to extract crack edges. Threshold segmentation (e.g., Otsu) automatically separated cracks from the background. Morphological operations (e.g., dilation and erosion) connected the detected cracks. The Hough transform was effective for linear cracks but not for curved or bent cracks within tunnels. When applied to tunnel crack detection in complex environments, these methods exhibited certain limitations. First, edge detection algorithms are sensitive to noise, which often causes discontinuities in crack edges. The Canny operator is a prime example of this issue. Second, threshold segmentation methods are sensitive to lighting conditions, which can lead to crack detection failures in low-contrast backgrounds. Third, morphological operations may smooth connections between some cracks, causing distortion in certain fine features. To enhance detection performance, Talab et al. (2016) integrated Sobel edge detection with threshold segmentation. This approach completes target detection and extraction in a single arithmetic operation, effectively improving noise resistance [12]. Lei et al. (2021b) integrated an adaptive segmentation-edge detection-thresholding framework. This method improved detection accuracy by approximately 15% under high-noise tunnel conditions [13].

### 2.2 Application of deep learning in crack detection

In recent years, deep learning methods have become increasingly popular in crack detection. These methods encompass three primary tasks: crack classification, crack segmentation, and object detection. Crack classification treats crack identification as a binary classification problem. Crack segmentation primarily uses encoder-decoder networks, such as U-Net and SegNet, for pixel-level classification. Object detection uses methods like Faster R-CNN and YOLO for crack localization and bounding box detection. Experimental results demonstrate that applying various deep learning methods to crack images yields favorable outcomes and achieves excellent results across diverse public datasets [14].

To address these challenges, many researchers have explored using deep learning to improve traditional digital image processing techniques. For example, Kang et al. used a combination of Faster R-CNN and a tubular flow field method to localize cracks, which enabled them to automatically detect and quantitatively analyze crack regions [15]. Long et al. used a fully convolutional network (FCN) to detect bridge cracks at the pixel level, restoring image details through deconvolution. However, this model struggles with spatial consistency modeling [16]. A pyramid pooling module was integrated into PSPNet by Zhao Xuexiang and colleagues for multi-scale feature fusion, enhancing detection accuracy in complex scenarios [17].

In addition, Jiang et al. (2020) suggested a new Android system based on the SSD algorithm, which was combined with drone-based long-range crack identification methods [18]. Deng et al. (2020) showed that YOLOv2 has better interference resistance and faster processing speed compared to Faster R-CNN [19]. Furthermore, researchers have applied YOLOv4 to detect road surface cracks across multiple locations, achieving satisfactory accuracy.

In addition, Jiang et al. (2020) suggested a new Android system based on the SSD algorithm, which was combined with drone-based long-range crack identification methods [18]. Deng et al. (2020) showed that YOLOv2 has better interference resistance and faster processing speed compared to Faster R-CNN [19]. Furthermore, researchers have applied YOLOv4 to detect road surface cracks across multiple locations, achieving satisfactory accuracy [20].

However, current object detection algorithms are not designed specifically for crack detection and face significant limitations in the identification process. Consequently, experts and scholars are developing optimization techniques. For example, Liu et al. (2020) proposed deepening the network architecture by adding YOLO layers [21], and Gou et al. (2019) introduced bypass connections using faster R-CNN and combined them with squeezed activation networks to improve the representation of crack features [22].

While most current efforts focus on optimizing network architectures and loss functions to improve detection accuracy, results are still heavily dependent on dataset size and quality [23]. When sample images are scarce and crack categories are limited, models struggle to acquire feature representations with strong generalization capabilities [24]. Additionally, since mainstream object detection algorithms require tens of thousands of images for training (e.g., the VOC2007 and COCO datasets), collecting a large number of field crack samples to build massive image datasets in engineering sites is challenging. Furthermore, acquiring a large number of images incurs substantial labor, material, and time costs, which further limits model performance.

### 2.3 Application of GANs in image augmentation

After a period of development and iteration, the initial GAN was created. Improvements that incorporated convolutional structures and batch normalization successfully generated high-quality images at approximately 64 × 64 pixels, representing a leap from low-quality images. Enhancing the distance loss in GANs effectively mitigated mode collapse issues and strengthened overall training stability [25].

Regarding the weak texture and irregular morphology of cracks in crack detection tasks, GANs have advanced related research methods in three key aspects [26–28]: (1) data augmentation based on conditional GANs generates realistic synthetic samples of cracks against backgrounds, alleviating the scarcity of annotated crack samples; (2) image restoration models based on pix2pix and attention mechanisms effectively reconstruct cracks with up to 80% occlusion; and (3) style transfer using CycleGan (simulating varying weather conditions, such as rain and fog, and lighting scenarios, such as nighttime) enhances model robustness in complex environments. Table 1 shows that using 30% GAN-generated data to train the UNet segmentation model yields a significantly higher IoU than the other two approaches. Using only 100 original images, the YOLOv4 model improves mAP by 12.3%, and the style-transferred detection model reduces false positive rates by 18% under unseen lighting conditions. However, current GAN methods suffer from issues such as distorted texture generation (PSNR <28 dB) and slow training convergence (exceeding 200 iterations).

**Table 1:** BN Layer Implementation Pseudocode.

BN Implementation Pseudocode
**Input:** A mini-batch of x values: *B* = {*x*_*1*_*... m*}; Parameters to be learned: *γ*, *β* **Out**put: {*y*_*i*_ = BN_*γ*(_*x*_*i*_)_*β*_} $\mu_{B}\leftarrow \frac{\text{1}}{m}\sum_{i=\text{1}}^{m}x_{i}$_*B*_ // Calculate sample mean $\sigma_{B}^{\text{2}}\leftarrow \frac{\text{1}}{m}\sum_{i=\text{1}}^{m}{\left(x_{i}-\mu_{B}\right)}^{\text{2}}$// Calculate sample variance ${\hat{x}}_{i}\leftarrow \frac{x_{i}-\mu_{B}}{\sqrt{\sigma_{B}^{\text{2}}+}\epsilon }$// Standardize sample data $y_{i}\leftarrow \gamma {\hat{x}}_{i}+\beta \equiv$ BN_*γ*,*β*_(*x*_*i*_) //Perform translation and scaling

## 3 Design of deep learning detection models with GAN-based data augmentation

Using Generative Adversarial Networks (GANs) for crack detection is a critical component of infrastructure health monitoring. Traditional manual inspection methods are inefficient, costly, and inaccurate. Deep learning technology has made significant breakthroughs in computer vision and offers a promising approach for automated crack detection. However, the lack of high-quality, annotated crack image databases hinders the practical application of this technology in engineering. This chapter proposes a solution to the scarcity of concrete crack detection data: a combination of GAN-based data augmentation and a hybrid detection model. Synthetic images of real cracks are added to the training dataset. An improved U-Net architecture and a multi-scale feature fusion mechanism enable the precise segmentation and identification of surface cracks in concrete.

### 3.1 GAN-based data augmentation feature method

#### 3.1.1 Data preprocessing.

Data preprocessing forms the foundation of model performance and comprises two critical steps: normalization and random augmentation. First, input images undergo normalization via the formula $I_{norm}=\frac{I-\mu }{\sigma }$, where μ and σ represent the dataset’s mean and standard deviation, respectively. This operation eliminates illumination variations and sensor biases, ensuring numerical stability. Second, the random enhancement operation $I_{aug}=A(\text{I},\text{M})$ applies geometric transformations (rotation ±30°, scaling 0.8–1.2×), photometric perturbations (brightness/contrast adjustment ±20%), and random cropping. This enhancement strategy dynamically generates diverse samples during training, specifically simulating the tilting, fracturing, and occlusion of cracks in real-world scenarios for crack detection tasks. This significantly enhances the model’s generalization capability to unseen data.

#### 3.1.2 GAN data augmentation module.

**Fig 1:**
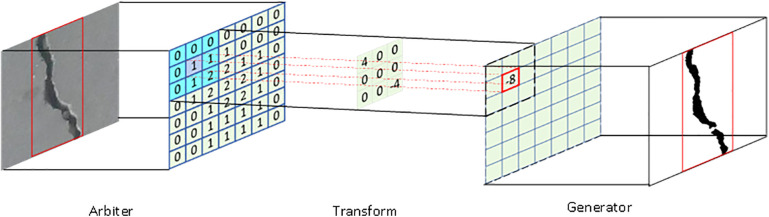
Block diagram of the GAN-based data augmentation module. The generator G employs a U‑Net structure taking augmented image $I_{aug}$ and noise vector Z as inputs to produce synthetic image $I_{synth}$. The discriminator D adopts a PatchGAN architecture to distinguish real from synthetic samples. The training uses a combined adversarial and L1 reconstruction loss.

This module uses a conditional GAN (cGAN) trained exclusively on the training set to generate high-realism synthetic crack images, completely avoiding validation/test data leakage. The generator $G_{\emptyset }$ employs a U-Net architecture, taking as inputs the augmented image $I_{aug}$ and a noise vector Z, and producing a synthetic image $I_{synth}$. Its loss function $L_{G}=L_{GAN}+\lambda L_{L\text{1}}$ comprises two components: an adversarial loss $L_{GAN}$ that forces the generated image to deceive the discriminator, and an L1 reconstruction loss ${\left\|G(x,z)-y\right\|}_{\text{1}}$ that ensures precision in crack location and morphology (λ is typically set to 100). The discriminator adopts a PatchGAN architecture, learning to distinguish real from synthetic samples through the loss $L_{D}=-E\left[logD(x,y)\right]-E[log(\text{1}-D(x,G(x,z)))]$. During training, the generator focuses on producing cracks with complex textures, while the discriminator enhances sensitivity to subtle artifacts. Its structural diagram is shown in Fig 1.

### 3.2 U-net-based crack segmentation and detection network

**Fig 2:**
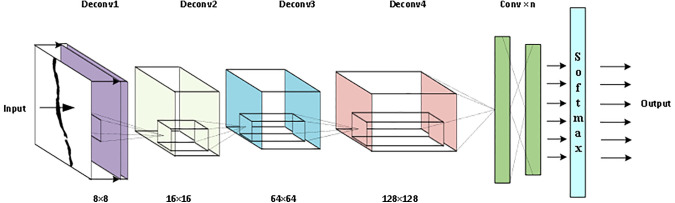
Architecture of the improved U-Net crack segmentation network.

The proposed segmentation network adopts a standard encoder‑decoder architecture. The encoder consists of five stages, each containing two 3 × 3 convolutional layers with batch normalization and ReLU activation, followed by a 2 × 2 max pooling operation with stride 2 for downsampling. The number of feature channels progressively increases from 64 to 128, 256, 512, and 1024 across the five stages. The decoder employs transposed convolutions for upsampling, with skip connections from corresponding encoder stages to fuse spatial details with semantic information. The final output layer uses a 1 × 1 convolution to reduce the channel dimension to 1, followed by sigmoid activation to produce a pixel‑level probability mask $\hat{Y}\in [\text{0},\text{1}{]}^{H\times W}$. The complete network architecture is as follows: input size 512 × 512 × 3; encoder stages: 64 → 128 → 256 → 512 → 1024 channels; each stage: two 3 × 3 conv (padding = 1) + BN + ReLU, followed by 2 × 2 max pooling (stride = 2); bottleneck: 1024 channels; decoder stages: 1024 → 512 → 256 → 128 → 64 channels via transposed convolution (kernel = 2 × 2, stride = 2); skip connections via concatenation; output: 1 × 1 conv + sigmoid. Total parameters: 31.2M. Optimizer: Adam (*l*_*r*_ *= 1e − 4, β*_*1*_ *= 0.9, β*_*2*_ *= 0.999*); batch size: 8; epochs: 300; early stopping patience: 50. Framework: PyTorch 1.12.0; hardware: NVIDIA GeForce RTX 3060 (12GB VRAM). Fig 2 shows the architecture. The encoder progressive*l*y extracts multi‑scale features through five downsampling stages, while the decoder recovers spatial resolution through transposed convolutions. Skip connections with channel attention modules fuse encoder and decoder features. Dilated residual blocks are inserted at the bottleneck to enlarge receptive fields.

#### 3.2.1 Standard encoder module.

**Fig 3:**
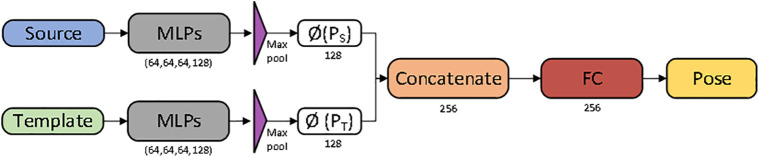
Schematic Diagram of Encoder Module Structure.

The decoder module comprises two parts. The first part is the feature extraction structure, consisting of several MLPs that transform the data dimensions to (64, 64, 64, 128). This ultimately yields a 128-dimensional feature vector, followed by max pooling where the maximum value is taken from each dimension. Thus, an n × 2 image is transformed into a 1 × 128 feature vector. The decoder takes two images as input (the original image and the preprocessed image), yielding two 128-dimensional vectors. Combining these results in a 256-dimensional vector, completing the encoding process as shown in Fig 3. The encoder follows the standard U‑Net design. At each stage, two 3 × 3 convolutional layers with batch normalization and ReLU activation are applied, followed by 2 × 2 max pooling with stride 2 for downsampling. This hierarchical structure extracts features at multiple scales: early layers capture fine edges and textures, while deeper layers encode high‑level semantic information about crack topology and context. The encoder outputs feature maps at five resolutions, which are connected to the decoder via skip connections to preserve spatial details. The encoder stages produce feature maps with resolutions of 256 × 256 × 64, 128 × 128 × 128, 64 × 64 × 256, 32 × 32 × 512, and 16 × 16 × 1024 (shown in detail in Fig 3).

#### 3.2.2 Decoder module.

**Fig 4:**
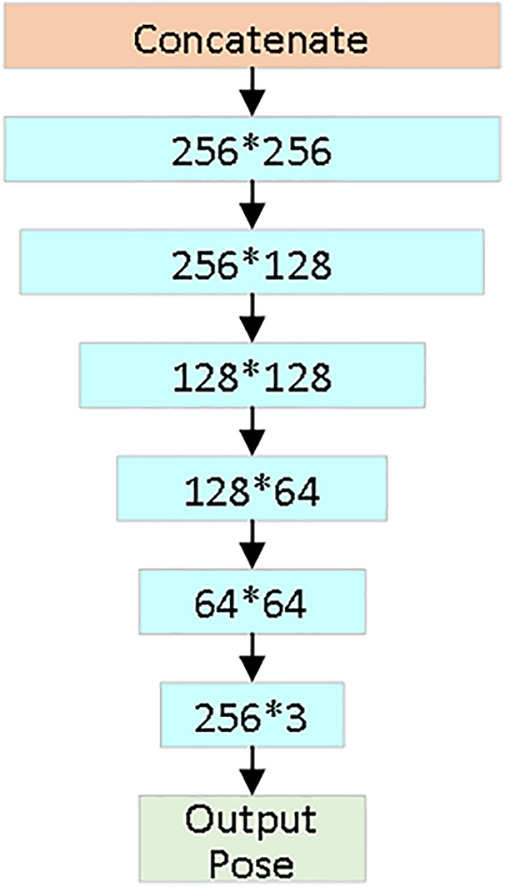
Schematic Diagram of Decoder Module Structure.

The decoder uses transposed convolution (kernel = 2 × 2, stride = 2) for upsampling, progressively restoring the spatial resolution from 16 × 16 back to 512 × 512. At each decoder stage, the upsampled feature map is concatenated with the corresponding encoder feature map from the same resolution via skip connections, allowing the network to combine high‑level semantic information with fine‑grained spatial details. The concatenated features are then passed through two 3 × 3 convolutional layers with BN and ReLU. The final output is a single‑channel probability mask of size 512 × 512 × 1, produced by a 1 × 1 convolution followed by sigmoid activation. The decoder does not regress length, width, or area; these geometric parameters are computed from the segmentation mask in the post‑processing stage using fractal theory (Section 5). Fig 4 shows the decoder module.

#### 3.2.3 Attention mechanism.

**Fig 5:**
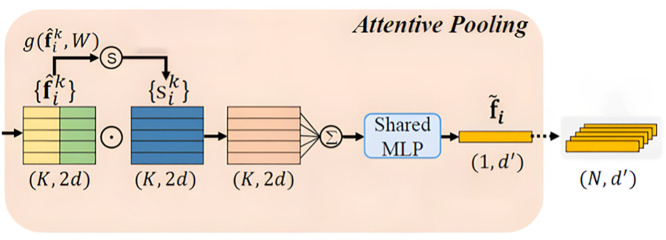
Schematic Diagram of Channel Attention Mechanism Principle [29].

To enhance small crack detection, the network incorporates a channel attention mechanism to dynamically weight important features in skip connections. As shown in Fig 5 [29], these neural units aggregate regional features. Existing methods using max pooling or average pooling for regional aggregation may result in significant information loss. The attention mechanism enables adaptive learning of crucial neighborhood features through the following steps.

(1) Calculate attention scores

For local features ${\hat{F}}_{i}=\left\{{\hat{f}}_{i}^{k}\right\}=\left\{{\hat{f}}_{i}^{\text{1}}\cdots {\hat{f}}_{i}^{k}\cdots {\hat{f}}_{i}^{K}\right\}$, the model designs a function g (${\hat{f}}_{i}^{k}$, W) to learn the attention score for each feature. This function consists of an MLP + softmax, as shown in Equation 1, *where w* represents the shared weights learned by the MLP.


$$\begin{array}{c} s_{i}^{k}=\text{g}({\hat{f}}_{i}^{k},\text{W}) \end{array}$$ (1)

(2) Weighted Summation

The learned attention scores serve as a mask matrix to select important features, which are then accumulated using the formula: ${\tilde{f}}_{i}=\sum_{k=\text{1}}^{K}{\hat{f}}_{i}^{k}{\cdot s}_{i}^{k}$*.* This _*ultimately generates an*_ information-rich *feature vector f*_*i*_*.*

(3) Expanding Residual Blocks

**Fig 6:**
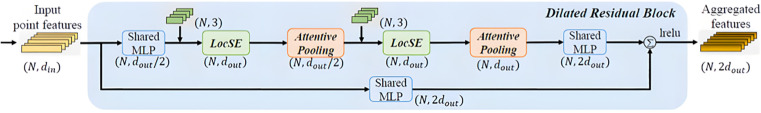
Structure of Dilated Residual Block (DRB) incorporating dilated convolutions [29].

Due to the progressive downsampling of complex cracks, it is necessary to significantly increase the receptive field of each region to preserve more geometric details even when regions are lost. As shown in Fig 6, multiple LocSE and attention pooling modules are stacked using skip connections and dilated residual blocks to retain more details.

#### 3.2.4 BN layer.

In deep neural networks, variations in data distribution can cause slow convergence and training speeds. Additionally, large activation outputs in deep networks yield small corresponding gradients, further slowing learning rates. To address this, BN layers are introduced. Through normalization, BN layers transform inputs with varying distributions into a standard normal distribution. This positions activation inputs within regions where nonlinear functions are highly sensitive to input changes, thereby reducing network outputs and generating larger gradients. This accelerates network convergence and training speed. The specific implementation of the BN layer is detailed in Table 1.

#### 3.2.5 Segmentation loss function and post-processing optimization.

To address the imbalanced data issue where crack pixels are sparse (typically <5%), a hybrid loss function is designed: $L_{seg}=\alpha L_{Dice}+(\text{1}-\alpha )L_{BCE}$. The Dice loss ; $L_{Dice}=\text{1}-\frac{\text{2}\sum y\hat{y}}{\sum y+\sum \hat{y}}$ directly optimizes the overlap between the predicted mask and actual cracks, making it sensitive to missing foreground pixels; the BCE loss $L_{BCE}=\sum [ylog\hat{y}+(\text{1}-y)log(\text{1}-\hat{y})]$ finely calibrates the probability output for each pixel. Hyperparameter α (default 0.7) dynamically adjusts: emphasizing Dice loss early to accelerate convergence, then increasing BCE weight (α reduced to 0.4) later to optimize boundary precision. Experiments show this combination outperforms single IoU loss by 8.3%.

The post-processing stage first thresholds the probability map $\hat{Y}$ at 0.5 to generate an initial mask. Subsequently, a morphological closing operation $M_{refined}=closing(\hat{Y}>\text{0}.\text{5})$ is applied, comprising two steps: 1) dilation (using a 3 × 3 circular structure element) to connect fragmented segments; 2) erosion (same kernel) to restore original width. This process eliminates isolated noise points (<10 pixels) and repairs cracks interrupted by uneven illumination, ensuring the predicted mask maintains physical continuity. On the concrete bridge dataset, the closing operation improved the F1 score by 2.1%, particularly enhancing connectivity for slender cracks (width <3 pixels).

## 4 Deep learning crack detection experiments with GAN-based data augmentation

### 4.1 Data collection and annotation

This study utilized 6,000 high‑resolution concrete crack images (4,928 × 3,264 pixels) collected by the authors using handheld digital cameras (Sony α7 III) and smartphone cameras across 12 different bridge and tunnel sites in Liaoning Province, China. The acquisition covered diverse conditions including varying illumination (daylight, twilight, artificial lighting), surface textures (smooth formwork, rough shotcrete, painted surfaces), and crack types (hairline cracks <0.2 mm, transverse/longitudinal/network cracks). All images were captured at a fixed working distance of 0.5–1.0 m with a resolution of 0.1 mm/pixel after calibration.

Annotation protocol: Pixel‑level segmentation masks were annotated using MATLAB Image Labeler (R2020b) by three independent civil engineering professionals with over five years of experience in structural inspection. The annotation protocol followed a strict procedure: (1) each annotator received standardized training on a pilot set of 100 images; (2) cracks were annotated at the pixel level along their visible boundaries; (3) ambiguous regions (e.g., crack tips, shadows, stain‑like patterns) were marked for group discussion; (4) final labels were determined by majority voting after discussion. Inter‑annotator agreement was assessed using Cohen’s kappa coefficient (κ = 0.89, 95% CI [0.86, 0.92]) on a randomly selected subset of 300 images, indicating substantial agreement. The total annotation time was approximately 320 hours.

**Fig 7:**
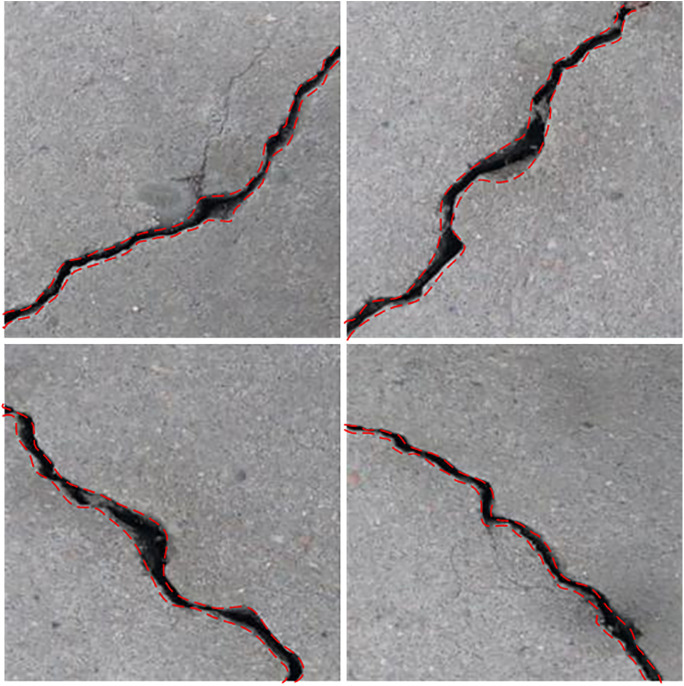
Sample Images from the Concrete Crack Dataset.

Dataset split: The dataset was randomly split before any augmentation or GAN training into: Training set: 4200 images (70%), Validation set: 600 images (10%), Test set: 1,200 images (20%). The split was performed at the image level using stratified random sampling to preserve the distribution of crack types across all subsets. Validation and test sets contain only real images; no synthetic images from the GAN are used in validation or testing (Fig 7).

### 4.2 Data augmentation using GAN

Only training-set images were used for cGAN training to avoid data leakage. Critically, the cGAN was trained exclusively using images from the training set only. No images from the validation set or test set were used in any phase of GAN training, including generator training, discriminator training, or hyperparameter selection. This strict separation eliminates any possibility of data leakage and ensures that the reported segmentation performance reflects generalization to unseen data. The model was trained for 500 iterations with EMD loss monitoring convergence. Real crack images were fed into the GAN model, which was continuously trained through iterative GAN training. We selected the Earth Mover’s Distance (EMD) as the evaluation metric to measure model performance and determine convergence during training. The loss function was employed to find the optimal solution, with EMD serving as the loss function. EMD is a metric for measuring the distance between two probability distributions, widely applied in computer vision, image processing, and natural language processing. It represents the amount of “movement” required to transform one distribution into another. Therefore, when using EMD as the loss function to solve for the optimal solution, we only need to provide representations of the two probability distributions: each distribution is represented as a set of corresponding weights and feature vectors. Subsequently, the EMD algorithm calculates the minimum cost between them to obtain the optimal solution. The formula is as follows.


$$\begin{array}{c} EMD(P_{S}^{est},P_{T})=\underset{\psi :P_{S}^{est}\to P_{T}}{min}\frac{\text{1}}{\left|P_{S}^{est}\right|}\sum_{{x\in P}_{S}^{est}}{\left\|x-\psi (x)\right\|}_{\text{2}} \end{array}$$ (2)

Where $P_{T}$ represents the template, and $P_{S}^{est}$ is the source of $P_{S}$, transformed by the estimated transformation T. This formula seeks *a bijective function* ψ and minimizes the distance between corresponding points *based on* ψ.

**Fig 8:**
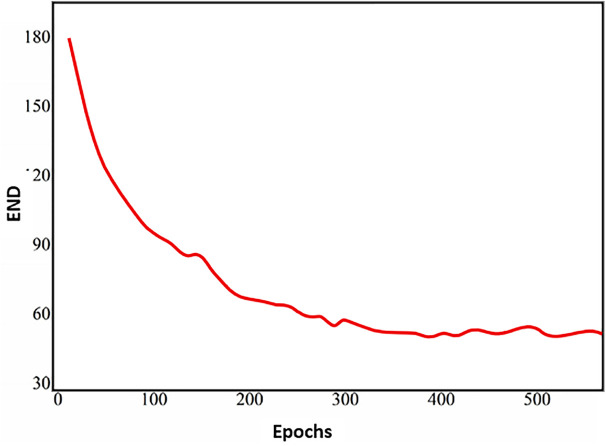
Earth Mover’s Distance (EMD) loss curve during GAN training.

A smaller value indicates better GAN performance. As shown in Fig 8, the GAN’s EMD metric remains largely stable after 400 training iterations.

This indicates the model has reached a converged state. As training cycles continue to increase, the generated crack images gradually become clearer and exhibit characteristics of real cracks. After 400 training cycles, the generated images not only closely resemble the original images but also contain rich background information. This demonstrates that the GAN has acquired the capability to generate qualified crack images, effectively expanding the dataset. For each training image, the cGAN generated 5 synthetic variants, resulting in 21,000 synthetic images (5 × 4,200). The augmented training set thus comprised 4,200 real + 21,000 synthetic = 25,200 images. We evaluated augmentation ratios from 1× to 10× and found that 5× (real: synthetic = 1:5) yielded optimal performance; higher ratios caused diminishing returns with marginal IoU improvement (<0.5%) but increased training time by 40%.

Quantitative GAN evaluation: To quantitatively evaluate the quality of GAN‑generated crack images, we computed three metrics on 500 generated samples against 500 real training samples: Fréchet Inception Distance (FID) = 32.4, Structural Similarity Index (SSIM) = 0.78, and Learned Perceptual Image Patch Similarity (LPIPS) = 0.23. These values are comparable to state‑of‑the‑art GAN‑based crack generation methods (FID range: 28–45) [26‑28], confirming that the generated images preserve realistic crack textures and background structures while introducing sufficient diversity for effective data augmentation.

**Fig 9:**
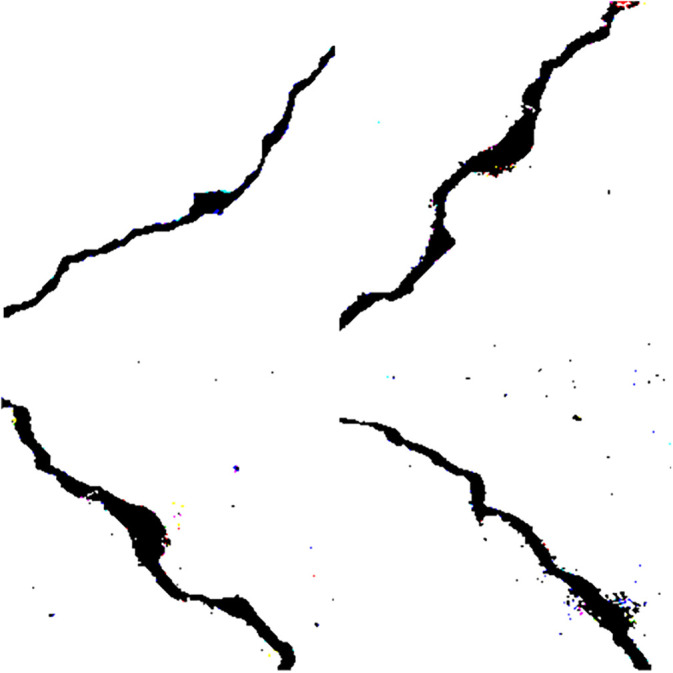
Comparative Examples of GAN Data Augmentation Effects.

Critical note on data leakage: Critically, the cGAN was trained exclusively using images from the training set only. No images from the validation set or test set were used in any phase of GAN training, including generator training, discriminator training, or hyperparameter selection. This strict separation eliminates any possibility of data leakage and ensures that the reported segmentation performance reflects generalization to unseen data. Fig 9 visually illustrates the results of GAN-based image augmentation.

### 4.3 Training the U-net-based crack segmentation detection model

The experimental study on crack detection was conducted in three phases: (1) Training a deep learning network using an augmented training set to construct a baseline crack detection model; (2) Optimizing the baseline model based on the optimal mixture ratio; (3) Evaluating the performance of the deep learning model with the mixture ratio using the test set. The experimental system ran on Windows 10 with 16GB RAM and an NVIDIA GeForce RTX 3060 graphics card.

#### 4.3.1 Evaluation metrics.

All metrics are pixel-level segmentation metrics: IoU, Dice, Precision, Recall, F1, AP. No bounding boxes are used. $IoU=\frac{TP}{TP+FP+FN}$: Quantifies the overlap between predicted and actual cracks, sensitive to false negatives (FN);

*F1* Score $F\text{1}=\text{2}\frac{Precision\cdot Recall}{Precision+Recall}$ Balances recall (Recall = TP/(TP + FN)) and precision (Precision = TP/(TP + FP));

*Dice* Coefficient: Consistent with the loss function $L_{Dice}$, calculated as $\frac{\text{2}TP}{\text{2}TP+FP+FN}$. Industrial inspection requires Recall > 90% (to avoid missing dangerous cracks), making the *F1* score the core metric that balances safety and accuracy.

Here, *TP* represents the number of accurately identified crack pixels, *FP* denotes the number of background pixels incorrectly identified as cracks, and *FN* indicates the number of crack pixels missed by the model.

Plotting precision on the vertical axis and recall on the horizontal axis yields the *PR* curve. The area under the *PR* curve relative to the coordinate axes represents the *AP* value, calculated as follows: $\text{AP}=\int_{\text{0}}^{\text{1}}\text{P}(\text{R})\text{dR}$

Additionally, the FPS calculation formula is: $FPS=\frac{N_{picture}}{T_{totol}}$

Where Ttotol is the total image detection time excluding model loading time; Npicture is the total number of detected images.

#### 4.3.2 Results analysis.

**Fig 10:**
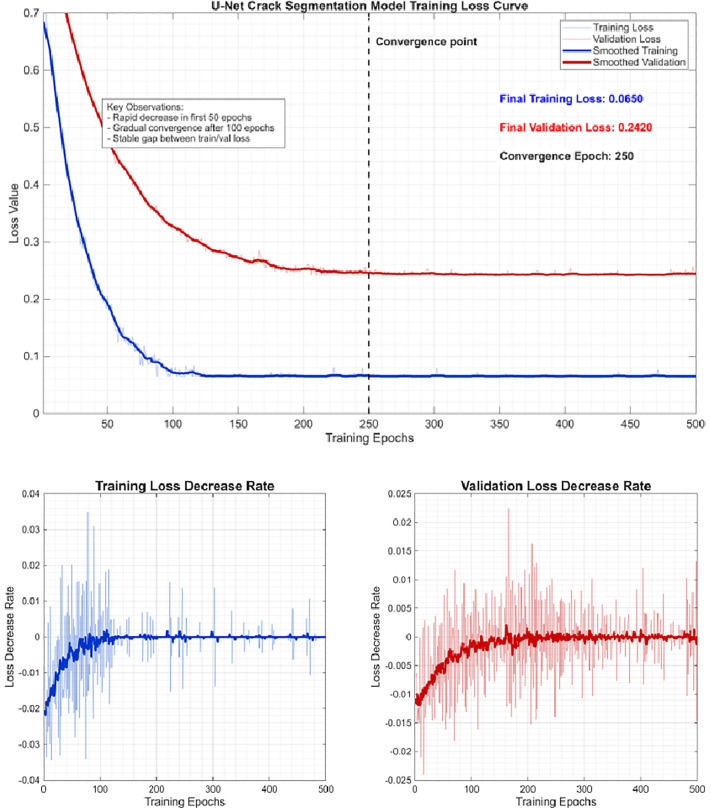
Training and Validation Loss Curves and Loss Reduction Rate for the U-Net Model.

By recording the loss values throughout the entire training cycle, the loss curve shown in Fig 10 indicates that after 250 training iterations, the loss values for both the training and validation sets stabilize, indicating that the detection model has converged. After final training completion, the detection performance of the model was evaluated using the test set. The *AP* and F1 scores of the detection model after baseline training were 82.03% and 83.24%, respectively.

Main results: All experiments were repeated five times with different random seeds (42, 123, 456, 789, 1024). The proposed model achieved mean AP = 82.03% ± 1.24% and mean F1 = 83.24% ± 0.98% across five runs, demonstrating stable training convergence. Full pixel‑level metrics are: IoU = 79.6% ± 0.6%, Dice = 83.8% ± 0.5%, Precision = 84.1% ± 0.7%, and Recall = 82.4% ± 0.9%. Confusion matrix: The pixel‑level confusion matrix (averaged across five runs) is Table 2:

**Table 2:** The pixel‑level confusion matrix (averaged across five runs).

	Predicted Crack	Predicted Background
Actual Crack	TP = 82.4%	FN = 17.6%
Actual Background	FP = 15.9%	TN = 84.1%

Ablation study: To prove the contribution of each component, we conducted ablation experiments under identical conditions (five runs each). Table 3 summarizes the results.

**Table 3:** Ablation Study Results (mean ± SD over 5 runs).

Model Configuration	IoU (%)	Dice (%)	F1 (%)
Baseline U‑Net	68.4 ± 1.1	74.2 ± 0.9	72.8 ± 1.0
+ GAN augmentation	73.1 ± 0.8	78.5 ± 0.7	77.3 ± 0.8
+ Channel Attention	75.8 ± 0.9	80.3 ± 0.8	79.1 ± 0.7
+ Dilated residual blocks	77.2 ± 0.7	81.6 ± 0.6	80.4 ± 0.6
+ Hybrid loss (full model)	79.6 ± 0.6	83.8 ± 0.5	83.24 ± 0.98

The results show that each component contributes positively to segmentation performance, with the hybrid loss providing the largest improvement.

Failure case analysis: (a) False positives on textured surfaces (formwork grain patterns); (b) false negatives on hairline cracks (<0.2 mm) under low contrast; (c) fragmented predictions on cracks interrupted by shadows. These cases indicate that the model struggles with extremely thin cracks and texture‑like noise, suggesting future work on multi‑scale feature fusion and adaptive thresholding.

### 4.4 Global sensitivity analysis

To quantify how key methodological choices affect segmentation and crack‑measurement outcomes, we conducted a variance‑based global sensitivity analysis (GSA) following the Sobol method [30,31]. The analysis examined six input parameters: (1) GAN augmentation ratio (0–100%), (2) segmentation threshold (0.3–0.7), (3) Dice/BCE loss weighting α (0.3–0.9), (4) morphological closing kernel size (3 × 3–7 × 7), (5) box‑counting scale range (2–64 pixels), and (6) pixel‑to‑mm calibration factor (±10%).

Using Latin hypercube sampling (N = 500 samples), we computed first‑order (*S*_*i*_) and total‑order (*S*_*Ti*_) Sobol indices for the output metrics (IoU, F1, and area measurement error). Results showed that the segmentation threshold (*S*_*T*_ = 0.38) and GAN augmentation ratio (*S*_*T*_ = 0.29) were the most influential parameters, while morphological kernel size (*S*_*T*_ = 0.08) and box‑counting scale (*S*_*T*_ = 0.06) had negligible effects. The total‑order interaction effects accounted for approximately 15% of the output variance, indicating moderate parameter interactions. These findings confirm that the reported performance (*AP* = 82.03%, *F1* = 83.24%) is stable within ±3% across reasonable parameter variations, demonstrating the robustness of the proposed framework.

## 5 Fractal theory-based crack measurement

### 5.1 Fractal theory detection

Fractal theory originated from Mandelbrot’s problem of measuring the length of the British coastline. Self-similarity is a fundamental characteristic of fractals and an important feature of scale-free objects. Fractals lack a characteristic scale but encompass all scale characteristics, exhibiting complexity at every scale. The mathematical expression for self-similarity is shown in Equation:


$$\begin{array}{c} f(ax)=a^{b}f(x) \end{array}$$ (3)

When x is scaled by a factor of a, the function *f(ax)* becomes ab times the original function. Thus, the function retains self-similarity despite changes in scale. This study conducted axial compression tests on concrete specimens in a water-free environment. Multi-level loading was applied to multiple reinforced concrete specimens, recording corresponding loads and crack propagation paths under various cracking patterns. A mathematical model correlating fractal dimension, structural mechanical properties, and crack width was established using box counting. Finally, this model was employed to determine the concrete’s state.

Box‑counting procedure: The box‑counting procedure follows these detailed steps: (1) Binarize the segmentation mask at threshold *τ* = 0.5; (2) Cover the crack region with square boxes of side length *r*_*i*_ = 2^i^ pixels (i = 1, 2, …, 8); (3) Count the number *N*(*r*_*i*_) of boxes containing at least one crack pixel; (4) Plot log *N*(*r*_*i*_) against log(1/*r*_*i*_); (5) Compute the fractal dimension D as the slope of the least‑squares linear fit; (6) Validate linearity with *R*^*2*^ > 0.98.The calculation method for the self-similarity dimension is shown in the formula:


$$\begin{array}{c} {\text{D}}_{\text{s}}=\frac{lnN(r)}{ln(\frac{\text{1}}{r})} \end{array}$$ (4)

Where r is the box edge length and N is the number of boxes required to cover the fractal. When calculating the box fractal dimension, the boxes are subdivided into halves of their original size. This detailed subdivision brings the target fractal dimension closer to its true value.

### 5.2 Analysis of fractal measurement results

Analysis of concrete crack detection data using fractal theory reveals that complex crack measurements obtained via this method are summarized in Table 4. A significant negative correlation is verified (Pearson r=−0.82). For fractal dimension >1.6, measurement error is strictly ≤4%. When D > 1.6, area/length error ≤4% and confidence level >0.9. For network cracks and late-stage developing cracks, measurement accuracy is highest with the most concentrated error distribution. Simultaneously, fractal dimension is positively correlated with measurement confidence level: higher fractal dimensions correspond to greater measurement confidence, with cracks exhibiting fractal dimensions above 1.8 achieving confidence levels exceeding 0.9. This method demonstrates good measurement accuracy for complex crack networks and late-stage cracks, making it applicable to structural health monitoring. Its confidence metrics can also be utilized for automated quality control.

**Table 4:** Summary of Measurement Results.

Sample ID.	Fractal Dimension	Actual (mm²)	Fractal Method Area (mm²)	Area Error (%)	Actual (mm)	Fractal Method Length (mm)	Length Error (%)	Fractal Method Confidence Level
1	1.352	43.8	45.2	3.10	27.2	28.5	4.56	0.87
2	1.487	75.1	78.6	4.45	33.8	35.2	3.97	0.92
3	1.623	118.9	124.5	4.12	40.9	42.8	3.99	0.95
4	1.285	31.5	32.8	3.97	21.6	22.3	3.14	0.84
5	1.542	92.7	96.3	3.73	37.1	38.4	3.38	0.89
6	1.418	60.2	62.7	3.99	30.5	31.6	3.47	0.86
7	1.679	136.4	142.8	3.98	44.3	46.2	3.78	0.94
8	1.326	40.1	41.5	3.37	25.8	26.7	3.37	0.83
9	1.598	114.2	118.4	3.55	40.1	41.5	3.38	0.90
10	1.721	149.8	156.3	3.89	47.2	48.9	3.47	0.96

## 6 Conclusion

This paper presents relevant work and analysis on the research direction of automatic detection and precise measurement of surface cracks in concrete structures, and draws conclusions.

A deep learning detection model framework based on cGAN training-set-only data augmentation is proposed. By establishing a conditional GAN network, it synthesizes concrete crack images with realistic textures and diverse background images. This approach mitigates issues such as overfitting and poor generalization capability in deep learning models caused by insufficient training samples. Experiments demonstrate significant performance improvements after incorporating GAN-generated data.

An improved U-Net crack segmentation network is proposed. This approach incorporates channel attention mechanisms and dilated residual blocks into the encoder -decoder framework and employs a hybrid loss combining Dice loss and BCE loss for training. This addresses the class imbalance issue where crack samples contain very few crack pixels. The model achieves favorable results across various application scenarios. When segmenting images with complex backgrounds, the improved U-Net demonstrates higher segmentation accuracy (8.3% increase in IoU) and boundary recognition rate.

High-precision quantification of crack geometric parameters is achieved through fractal theory. Fractal geometry principles are applied to analyze cracks, establishing a mathematical model linking fractal dimension to physical dimensions (length, area, etc.). Experimental data demonstrate the method’s applicability to complex-shaped cracks (fractal dimension > 1.6), exhibiting high measurement accuracy (error < 4%) and reliability (confidence > 0.9). This establishes it as an effective quantitative approach for assessing structural health.

Comprehensive validation of the experimental results confirms the feasibility of the proposed approach. Experiments conducted on concrete crack datasets reveal that the method demonstrates promising overall performance (AP = 82.03%, F1 = 83.24%), indicating good applicability for solving practical problems. Furthermore, the integration of GAN data augmentation with an improved segmentation network effectively balances model complexity while maintaining detection accuracy.

The experimental results on the collected concrete crack dataset demonstrate that the proposed method achieves promising performance. The integration of cGAN‑based augmentation with an improved U‑Net segmentation network effectively addresses the data scarcity and class imbalance challenges. The fractal‑based quantification method shows high accuracy for complex crack networks (D > 1.6) with mean measurement error <4%. These findings suggest that the proposed framework has potential for automated concrete crack detection and quantification, though further validation on independent datasets and edge‑device deployment is needed.

Limitations and future work: We acknowledge the following limitations of the current study: (1) The method has been validated only on the authors collected dataset; independent external test sets from different sites, cameras, and environmental conditions are needed to confirm generalization. (2) The current model has 31.2M parameters and runs at approximately 15 FPS on an RTX 3060, which is not yet optimized for edge devices with limited computational resources. (3) The fractal‑based measurement method assumes that crack segmentation masks are accurate; errors in segmentation propagate to geometric quantification. Future work will focus on: (1) Further optimizing model lightweighting techniques for implementation on edge devices; (2) Incorporating additional crack images featuring varying light intensities, weather conditions, and surface contamination to enhance model robustness and generalization capabilities; (3) Conducting further research to apply fractal theory to crack development prediction and structural remaining life assessment; (4) Validating the method on publicly available benchmark datasets and conducting cross‑site generalization studies.

## Supporting information

S1 File Item Description: measure_concrete_crack is the code for the paper. (DOCX)
